# Anti-PAR2 autoantibodies are associated with chronic histological injury in kidney transplant recipients

**DOI:** 10.3389/fimmu.2026.1923916

**Published:** 2026-08-20

**Authors:** Karolina Marek-Bukowiec, Jakub Mizera, Harald Heidecke, Kai Schulze-Forster, Łucja Janek, Ignacy Tarski, Mateusz Minor, Guido Moll, Rusan Ali Catar, Patryk Jerzak, Magdalena Kuriata-Kordek, Piotr Donizy, Agnieszka Hałoń, Krzysztof Kujawa, Dariusz Janczak, Mirosław Banasik

**Affiliations:** 1 Department of Nephrology, Transplantation Medicine and Internal Diseases, Institute of Internal Diseases, Wroclaw Medical University, Wroclaw, Poland; 2 Clinical Department of Nephrology, Transplantation Medicine and Internal Diseases, Institute of Internal Diseases, University Clinical Hospital in Wroclaw, Wroclaw, Poland; 3 CellTrend GmbH, Luckenwalde, Germany; 4 Statistical Analysis Centre, Wroclaw Medical University, Wroclaw, Poland; 5 Division of Histology and Embryology, Department of Human Morphology and Embryology, Faculty of Medicine, Wroclaw Medical University, Wroclaw, Poland; 6 Berlin Institute of Health (BIH) Center and School for Regenerative Therapies (BCRT/BSRT), Charité Universitätsmedizin Berlin, Corporate Member of Freie Universität Berlin, Humboldt-Universität zu Berlin, and Berlin Institute of Health (BIH), Berlin, Germany; 7 Julius Wolff Institute (JWI), Charité Universitätsmedizin Berlin, Corporate Member of Freie Universität Berlin, Humboldt-Universität zu Berlin, and Berlin Institute of Health (BIH), Berlin, Germany; 8 Department of Nephrology and Internal Intensive Care Medicine, Charité Universitätsmedizin Berlin, Corporate Member of Freie Universität Berlin, Humboldt-Universität zu Berlin, and Berlin Institute of Health (BIH), Berlin, Germany; 9 Department of Clinical and Experimental Pathology, Wroclaw Medical University, Wroclaw, Poland; 10 Department of Vascular, General, and Transplant Surgery, Wroclaw Medical University, Wroclaw, Poland

**Keywords:** anti-PAR1 autoantibodies, anti-PAR2 autoantibodies, chronic allograft injury, GPCR autoantibodies, kidney transplantation, non-HLA antibodies

## Abstract

**Introduction:**

Non-HLA autoantibodies directed against G protein-coupled receptors (GPCRs) have emerged as potential contributors to endothelial activation, vascular injury, rejection, and chronic allograft dysfunction after kidney transplantation. Protease-activated receptors 1 and 2 (PAR1 and PAR2) are GPCRs located at the interface of coagulation, inflammation, endothelial signaling, and tissue remodeling. However, the clinical relevance of circulating anti-PAR1 and anti-PAR2 autoantibodies in kidney transplant recipients remains undetermined.

**Methods:**

We performed a retrospective observational study including 123 kidney transplant recipients who underwent clinically indicated allograft biopsy between 2018 and 2025. The primary objective was to evaluate associations between serum anti-PAR1 and anti-PAR2 autoantibody concentrations and the severity of Banff histopathological lesions. Associations with rejection phenotypes, selected clinical variables, and graft outcomes were also evaluated. Antibody concentrations were measured by ELISA. Correlation analyses, non-parametric group comparisons, logistic regression models, and exploratory landmark analyses of death-censored graft survival from serum sampling were performed. Correction for multiple testing was applied using the Benjamini–Hochberg false discovery rate procedure.

**Results:**

Anti-PAR2 concentrations showed weak but statistically significant associations with interstitial fibrosis (Kendall τ = 0.221, pFDR = 0.005), tubular atrophy (Kendall τ = 0.216, pFDR = 0.005), arteriolar hyalinosis (Kendall τ = 0.193, pFDR = 0.018), and total inflammation (Kendall τ = 0.226, pFDR = 0.005). Borderline associations were observed for peritubular capillaritis, transplant glomerulopathy, and inflammation in areas of interstitial fibrosis and tubular atrophy (all pFDR = 0.052). No histopathological associations remained significant for anti-PAR1. Both anti-PAR1 and anti-PAR2 concentrations varied with time from transplantation to biopsy, while anti-PAR2 was additionally associated with recipient age at transplantation. Neither antibody was significantly associated with rejection phenotypes. In the exploratory landmark analysis, anti-PAR2 concentrations above the cohort median were associated with a higher hazard of death-censored graft loss (HR = 2.136, 95% CI 1.202–3.795; p = 0.010).

**Discussion:**

Higher anti-PAR2 autoantibody concentrations were weakly associated with several chronic histological lesion scores in kidney allograft biopsies. An exploratory landmark analysis showed poorer death-censored graft survival among recipients with anti-PAR2 concentrations above the cohort median, although this association was not significant when anti-PAR2 was analyzed continuously. These findings require prospective validation.

## 1 Introduction

Kidney transplantation is the preferred treatment for end-stage kidney disease, but long-term graft survival remains limited by progressive allograft injury. Despite major advances in immunosuppressive therapy and post-transplant monitoring, chronic allograft dysfunction remains a leading cause of kidney allograft failure after the first post-transplant year. Although donor-specific anti-HLA antibodies (DSA) are essential mediators of antibody-mediated rejection (AMR) and microvascular injury (MVI), they do not fully explain the heterogeneity of graft damage observed in clinical practice. A growing body of evidence indicates that non-HLA autoimmune responses may contribute to endothelial injury, vascular inflammation, chronic rejection-like phenotypes, and progressive structural remodeling of the kidney allograft (1, 2).

Among non-HLA targets, functional autoantibodies directed against G protein-coupled receptors (GPCRs) have received particular attention. Antibodies against the angiotensin II type 1 receptor (AT1R) and endothelin A receptor (ETAR) are the most extensively studied examples and have been associated with vascular rejection phenotypes, microvascular inflammation, and inferior transplant outcomes (3, 4). Previous studies from our center also supported the clinical relevance of GPCR-directed non-HLA antibodies in kidney transplantation. Anti-AT1R antibodies were associated with poorer early graft function and an increased risk of graft loss, whereas anti-ETAR antibodies were linked to impaired graft function during the first post-transplant year (5, 6). Combined analyses of anti-AT1R and anti-ETAR antibodies further suggested their association with morphological and functional allograft injury and graft loss (7). In addition, biopsy-based studies indicated that ETAR expression within kidney allograft compartments may be associated with antibody-mediated injury (8–10). More recent analyses extended this concept to microvascular inflammation (11). Together, these observations support the concept that receptor-directed autoantibodies may be clinically relevant and, in some settings, biologically active, even in the absence of conventional HLA donor-specific antibodies. Nevertheless, AT1R and ETAR antibodies do not account for the full spectrum of non-HLA-mediated injury, indicating that additional GPCR-directed responses may be involved.

Protease-activated receptors (PARs) constitute a distinct subfamily of GPCRs activated by proteolytic cleavage. PAR1 is activated predominantly by thrombin and participates in platelet activation, coagulation, endothelial activation, inflammatory signaling, and vascular responses. PAR2 is activated by several serine proteases and has been implicated in chronic inflammation, epithelial injury, fibroblast activation, extracellular matrix deposition, and tissue remodeling (12, 13). In renal disease models, PAR signaling has been linked to glomerular and tubular injury, diabetic kidney disease, acute kidney injury, nephrotoxicity, and renal fibrosis (12, 13).

PAR1-directed autoimmunity has recently been linked to endothelial activation in systemic and transplant-related vascular disease. Immunoglobulin G fractions from patients with kidney allograft vasculopathy were shown to induce a proinflammatory endothelial switch mediated through PAR1-dependent TNF-alpha signaling and subsequent monocyte activation (14). Similar mechanistic observations were reported in scleroderma renal crisis, where patient-derived IgG stimulated endothelial IL-6 and endothelin-1 production through PAR1-dependent pathways (15, 16). These studies support the biological plausibility of PAR-directed autoantibody effects on vascular and endothelial compartments.

The role of circulating anti-PAR1 and anti-PAR2 autoantibodies in kidney transplantation remains largely unexplored. This is particularly relevant because PAR-dependent signaling links coagulation, endothelial activation, inflammation, epithelial injury, and tissue remodeling, processes that may contribute to different patterns of kidney allograft damage. The present study therefore evaluated associations between circulating anti-PAR1 and anti-PAR2 autoantibody concentrations and the severity of individual Banff histopathological lesions in kidney transplant recipients undergoing clinically indicated allograft biopsy. Associations with rejection phenotypes, selected clinical characteristics, and graft outcomes were also assessed.

## 2 Materials and methods

### 2.1 Study design and population

The study was conducted in accordance with the Declaration of Helsinki, and the study protocol was approved by the local Bioethics Committee of Wroclaw Medical University (approval IDs: KB 30/2025 and 479/2025).

This retrospective observational study included 123 adult kidney transplant recipients aged ≥18 years who underwent clinically indicated kidney allograft biopsy between 2018 and 2025. Patients were included if serum samples and histopathological biopsy data were available. Serum samples used for anti-PAR1 and anti-PAR2 antibody measurements were collected at the time of biopsy or within a median interval of 2 days from biopsy (Q1-Q3 1–29 days). Clinical data were extracted from medical records and included recipient sex, recipient age at transplantation, donor type, number of transplantations, time from transplantation to biopsy, follow-up time, graft loss, and rejection diagnoses. For the exploratory graft survival analyses, follow-up was calculated from the date of the serum sampling. Death-censored graft loss was defined as the event of interest, whereas death with a functioning graft was treated as a censoring event at the date of death. The primary landmark analysis included recipients with a functioning graft and a follow-up time greater than zero after the serum sampling. A sensitivity analysis additionally included five recipients with no positive follow-up time after the index date, for whom graft loss was coded at time zero.

### 2.2 Histopathological assessment

Kidney allograft biopsies were evaluated according to the Banff classification criteria applicable at the time of biopsy assessment between 2018 and 2025. No histopathological re-assessment was performed specifically for the purposes of this study. The following Banff lesion scores were extracted and analyzed: interstitial inflammation (i), tubulitis (t), intimal arteritis (v), glomerulitis (g), peritubular capillaritis (ptc), interstitial fibrosis (ci), tubular atrophy (ct), fibrous intimal thickening (cv), transplant glomerulopathy (cg), mesangial matrix increase (mm), arteriolar hyalinosis (ah), total inflammation (ti), inflammation in areas of interstitial fibrosis and tubular atrophy (i-IFTA), and C4d deposition in peritubular capillaries. Microvascular inflammation (MVI) was defined as the sum of the Banff glomerulitis and peritubular capillaritis scores (g + ptc). For categorical analyses, MVI positivity was defined as g + ptc ≥ 2. Rejection phenotypes included antibody-mediated rejection (AMR), T-cell-mediated rejection (TCMR), borderline changes suspicious for TCMR, mixed rejection, and no rejection. Most Banff lesion scores had limited missingness; the largest number of missing observations was observed for mesangial matrix expansion.

### 2.3 Measurement of anti-PAR1 and anti-PAR2 autoantibodies

Serum anti-PAR1 and anti-PAR2 autoantibody concentrations were measured in duplicate using enzyme-linked immunosorbent assay (ELISA) kits and expressed as U/mL. The analyses were performed by CellTrend GmbH (Luckenwalde, Germany). No predefined positivity cut-off was applied. Antibody concentrations were analyzed primarily as continuous variables. For the exploratory graft survival analyses, concentrations were additionally dichotomized at the cohort medians: 5.33 U/mL for anti-PAR1 and 4.79 U/mL for anti-PAR2.

### 2.4 Statistical analysis

Statistical analyses were performed in R version 4.5.3 using the packages stats, survival, survminer, rstatix, car, and ggplot2 (**Supplementary Table 1**). Continuous variables were summarized as medians and interquartile ranges, whereas categorical variables were presented as counts and percentages. Distributional assumptions were assessed using the Shapiro–Wilk test and visual inspection of Q–Q plots (**Supplementary Figure 1**, **Supplementary Table 2**). Because most variables were non-normally distributed, non-parametric methods were used throughout.

Associations between antibody concentrations and individual Banff lesion scores constituted the primary analyses. Analyses of rejection phenotypes and selected clinical characteristics were considered secondary, whereas graft survival analyses and additional regression models were exploratory.

The association between anti-PAR1 and anti-PAR2 concentrations was assessed using Spearman rank correlation. Associations between antibody concentrations and ordinal histopathological or clinical variables were evaluated using Kendall rank correlation coefficients because of the high proportion of tied ranks in Banff lesion scores and selected clinical variables (**Supplementary Tables 3**, **4**). Correction for multiple testing was performed using the Benjamini–Hochberg false discovery rate procedure.

Between-group comparisons were performed using the Mann–Whitney U test for two groups and the Kruskal–Wallis test for more than two groups. When the Kruskal–Wallis test was statistically significant, Dunn *post hoc* testing with Holm correction was applied.

Exploratory death-censored kidney allograft survival analyses were performed using a landmark design, with the date of serum sampling as the analytical time origin. Graft loss was treated as the event of interest. Recipients without graft loss were censored at the last available follow-up or at death with a functioning graft. The primary landmark analysis included recipients with a functioning graft and a follow-up time greater than zero after the index date. A sensitivity analysis additionally included five recipients with no positive follow-up time after the index date, for whom graft loss was coded at time zero. Kaplan–Meier curves and log-rank tests were used to compare recipients with antibody concentrations above and below the cohort medians (**Supplementary Figure 2**). Univariable Cox proportional hazards regression models were fitted with anti-PAR1 and anti-PAR2 concentrations analyzed both continuously and after median-based dichotomization. The proportional hazards assumption was assessed using scaled Schoenfeld residuals. For continuous antibody concentrations, the adequacy of the linear functional form was evaluated using Martingale residual plots and by comparing linear Cox models with models incorporating natural cubic splines with three degrees of freedom using likelihood ratio tests (**Supplementary Figure 2**, **Supplementary Table 5**).

Logistic regression models were used to evaluate associations with graft rejection and the AMR component. Antibody concentrations were analyzed as continuous variables. For AMR component analyses, model comparison was performed using the likelihood ratio test and Akaike information criterion to determine whether adding anti-PAR2 to a model containing anti-PAR1 improved model fit. Influential observations and linearity of the logit were assessed using Cook distance and LOESS plots, respectively, and are presented in **Supplementary Figure 3**. Multicollinearity and model discrimination were evaluated using variance inflation factors and the concordance index, respectively, with the results reported in **Supplementary Table 8**.

All tests were two-sided, and a p-value below 0.05 was considered statistically significant.

## 3 Results

### 3.1 Baseline characteristics of kidney transplant recipients

Baseline characteristics of the study cohort are shown in **Table 1**. The study included 123 adult kidney transplant recipients. During the overall follow-up period, graft loss occurred in 60 patients (48.8%). The median time from transplantation to blood collection was 3.79 years (Q1-Q3 0.43-8.38). Pure AMR was identified in only three patients; therefore, rejection phenotypes were analyzed using component-based definitions. The AMR component, including pure AMR and mixed AMR/TCMR cases, was identified in 23 patients (18.7%). A TCMR component, including borderline changes suspicious for TCMR, pure TCMR, and mixed AMR/TCMR cases, was identified in 77 patients (62.6%). The median serum anti-PAR1 concentration was 5.33 U/mL (Q1-Q3 3.48-8.19 U/mL), and the median anti-PAR2 concentration was 4.79 U/mL (Q1-Q3 3.00-8.22 U/mL). The observed concentration ranges were 0.48–53.89 U/mL for anti-PAR1 and 0.15–49.06 U/mL for anti-PAR2.

**Table 1:** Characteristics of the study cohort.

Parameter	Value
Number of patients	123
Female sex, n (%)	45 (36.6%)
Male sex, n (%)	78 (63.4%)
Recipient age at transplantation, years, median (Q1-Q3)	37 (28-48)
Time from transplantation to biopsy, years, median (Q1-Q3)	3.78 (0.49-8.48)
Time from transplantation to blood collection, years, median (Q1-Q3)	3.79 (0.43-8.38)
Follow-up from transplantation to graft loss or end of observation, years, median (Q1-Q3)	6.02 (2.93-9.93)
Time from serum sampling to last observation, years, median (Q1-Q3)	1.64 (0.67–3.02)
Anti-PAR1 concentration, U/mL, median (Q1-Q3)	5.33 (3.48-8.19)
Anti-PAR2 concentration, U/mL, median (Q1-Q3)	4.79 (3.00-8.22)
Living donor, n (%)	19/121 (15.7%)
Deceased donor, n (%)	102/121 (84.3%)
Graft loss, n (%)	60 (48.8%)
AMR component, n (%)	23 (18.7%)
TCMR component, n (%)	77 (62.6%)

Q1–Q3, first and third quartiles.

The primary landmark survival analysis included 117 recipients with a functioning graft and a positive follow-up time after serum sampling. A total of 54 death-censored graft-loss events occurred in this cohort. The sensitivity analysis additionally included five recipients with no positive follow-up time after the index date, for whom graft loss was coded at time zero, resulting in 122 recipients and 59 events.

### 3.2 Relationship between anti-PAR1 and anti-PAR2 autoantibody levels

**Figure 1:**
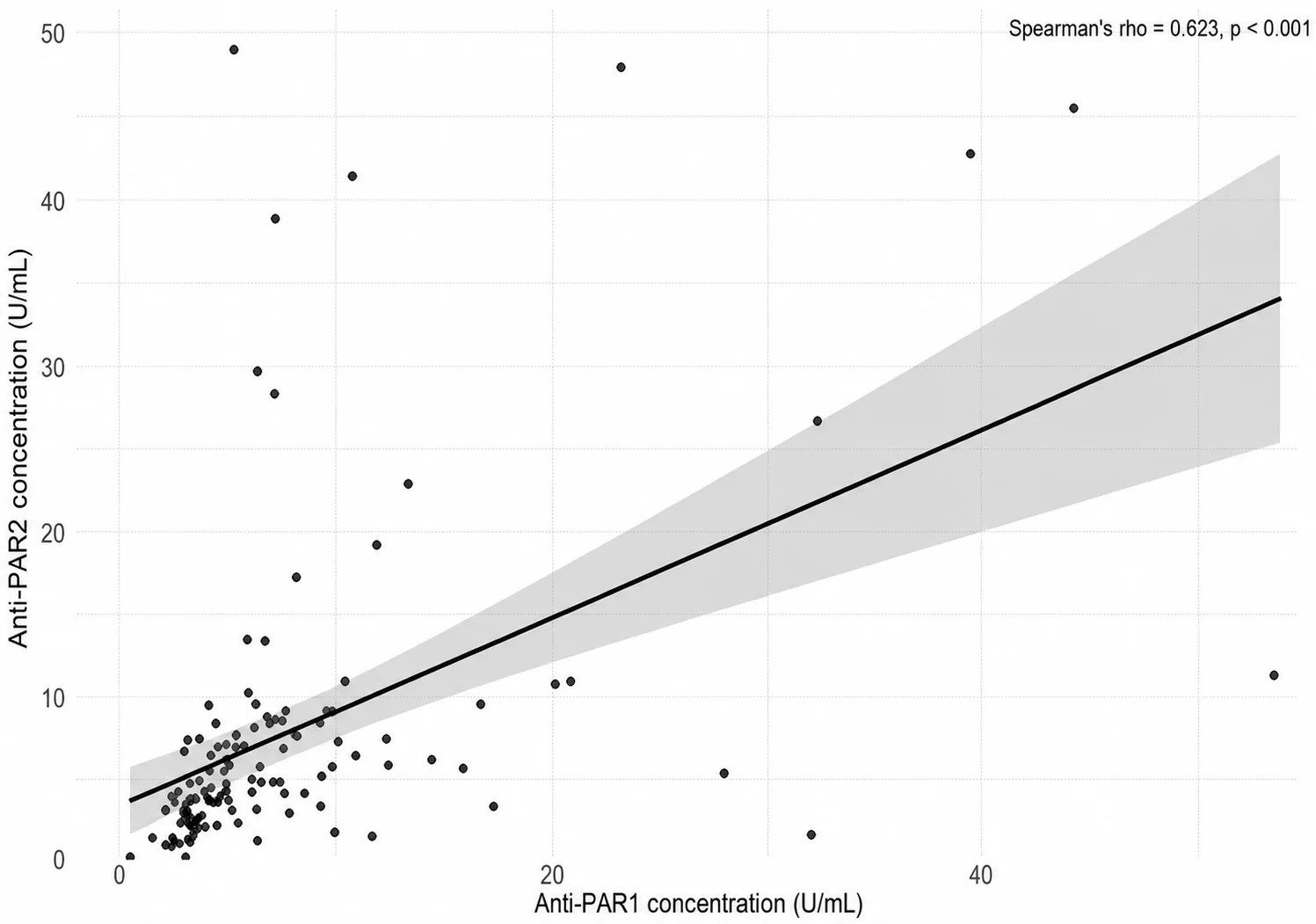
Spearman correlation between serum anti-PAR1 and anti-PAR2 autoantibody concentrations in 123 kidney transplant recipients. Shaded area represents the 95% confidence interval.

Anti-PAR1 and anti-PAR2 levels demonstrated a significant positive correlation (Spearman’s ρ = 0.623, p < 0.001; n = 123) (**Figure 1**). This indicates that recipients with higher anti-PAR1 levels tended to have higher anti-PAR2 levels, suggesting partial overlap in PAR-directed humoral responses.

### 3.3 Anti-PAR autoantibody levels in relation to Banff histopathological lesions

**Figure 2:**
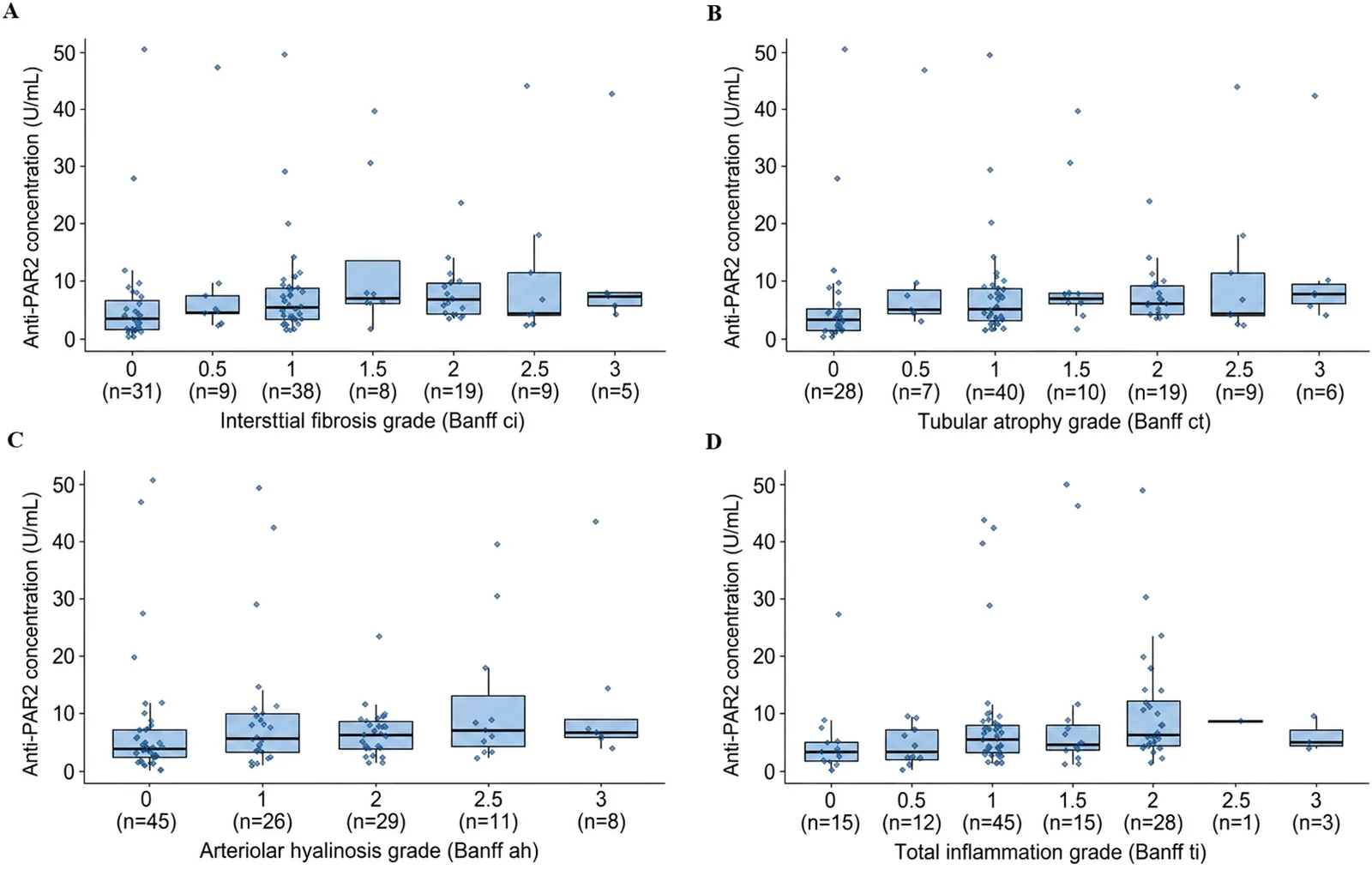
Serum anti-PAR2 autoantibody concentrations according to selected Banff histopathological lesion scores. Boxplots show anti-PAR2 concentrations across the recorded grades of **(A)** interstitial fibrosis (ci), **(B)** tubular atrophy (ct), **(C)** arteriolar hyalinosis (ah), and **(D)** total inflammation (ti). Boxes represent the interquartile range, horizontal lines indicate medians, whiskers extend to 1.5 times the interquartile range, and individual observations are shown as jittered points. Group sizes are provided below each category. Intermediate half-point values resulted from averaging the uncertain histopathological assessments.

The main histopathological finding was a weak but consistent association between higher anti-PAR2 concentrations and greater severity of several chronic allograft injury lesions. The distributions of anti-PAR2 concentrations across the recorded grades of interstitial fibrosis, tubular atrophy, arteriolar hyalinosis, and total inflammation are shown in **Figure 2**.

Anti-PAR2 concentrations were positively correlated with the severity of interstitial fibrosis (ci; τ = 0.221, pFDR = 0.005), tubular atrophy (ct; τ = 0.216, pFDR = 0.005), arteriolar hyalinosis (ah; τ = 0.193, pFDR = 0.018), and total inflammation (ti; τ = 0.226, pFDR = 0.005). Suggestive borderline associations were also observed for peritubular capillaritis, transplant glomerulopathy, and inflammation in areas of interstitial fibrosis and tubular atrophy (i-IFTA**;** all pFDR = 0.052).

In contrast, no association between anti-PAR1 concentrations and individual Banff lesion scores remained statistically significant after FDR correction (**Table 2**). The effect sizes of the statistically significant anti-PAR2 associations were modest, with Kendall’s τ values ranging from approximately 0.19 to 0.23.

**Table 2:** Correlations between anti-PAR1 and anti-PAR2 concentrations and Banff lesion scores.

Histopathological parameter	anti-PAR1 τ	p	pFDR	anti-PAR2 τ	p	pFDR
Interstitial inflammation (i)	0.041	0.543	0.760	0.047	0.485	0.617
Tubulitis (t)	-0.008	0.907	0.939	-0.017	0.799	0.799
Arteritis (v)	0.010	0.892	0.939	0.020	0.786	0.799
Glomerulitis (g)	-0.005	0.939	0.939	-0.033	0.635	0.741
Peritubular capillaritis (ptc)	0.102	0.136	0.389	0.153	0.026	**0.052**
Interstitial fibrosis (ci)	0.115	0.088	0.389	0.221	0.001	**0.005**
Tubular atrophy (ct)	0.129	0.056	0.389	0.216	0.001	**0.005**
Fibrous intimal thickening (cv)	0.034	0.647	0.823	0.056	0.448	0.617
Transplant glomerulopathy (cg)	0.074	0.318	0.620	0.172	0.020	0.052
Mesangial matrix increase (mm)	-0.056	0.465	0.723	-0.084	0.277	0.431
Arteriolar hyalinosis (ah)	0.102	0.139	0.389	0.193	0.005	**0.018**
Total inflammation (ti)	0.146	0.033	0.389	0.226	<0.001	**0.005**
i-IFTA	0.073	0.297	0.620	0.157	0.024	0.052
C4d deposition	0.062	0.354	0.620	0.078	0.248	0.431

Kendall’s τ coefficients are shown. pFDR values were adjusted using the Benjamini–Hochberg false discovery rate procedure. i-IFTA, inflammation in areas of interstitial fibrosis and tubular atrophy.

Bold values indicate statistically significant associations after FDR correction (pFDR < 0.05).

Because several higher-grade categories contained small numbers of observations, the boxplots are intended as descriptive visualizations, whereas formal inference was based on Kendall rank correlations.

### 3.4 Anti-PAR autoantibodies and rejection phenotypes

Anti-PAR1 and anti-PAR2 concentrations did not differ significantly according to rejection-related phenotypes. For microvascular inflammation, the Mann–Whitney U statistics were 1677 for anti-PAR1 (p = 0.520) and 1587 for anti-PAR2 (p = 0.265). Similarly, no significant differences were observed according to the presence of an AMR component, with U = 848 for anti-PAR1 (p = 0.135) and U = 897 for anti-PAR2 (p = 0.246), or according to the presence of a TCMR component, with U = 1722 for anti-PAR1 (p = 0.718) and U = 1828 for anti-PAR2 (p = 0.347). Detailed numerical results are provided in **Supplementary Table 6**.

Univariable logistic regression likewise showed no significant association between either antibody and graft rejection. Anti-PAR1 showed an OR of 1.017 per 1 U/mL increase (95% CI, 0.962–1.067; p = 0.499), whereas anti-PAR2 showed an OR of 1.023 (95% CI, 0.979–1.065; p = 0.262). Adding anti-PAR2 to a model containing anti-PAR1 did not improve prediction of the AMR component (likelihood ratio test χ² = 0.76, df = 1, p = 0.384), and neither antibody was significantly associated with the AMR component in the combined two-antibody model. The sensitivity analysis after exclusion of influential observations yielded consistent results (**Supplementary Table 7**).

Detailed comparisons across individual histopathological rejection diagnoses are presented in **Supplementary Tables 9** and **10**. These analyses should be interpreted cautiously because the cohort included only 23 recipients with an AMR component and only three recipients with isolated AMR.

### 3.5 Anti-PAR autoantibodies in relation to clinical characteristics and post-transplant timing

Anti-PAR2 concentrations were weakly and inversely correlated with recipient age at transplantation (τ = −0.177, pFDR = 0.005) and positively correlated with time from transplantation to biopsy (τ = 0.310, pFDR = 0.001; n = 123). Anti-PAR1 concentrations were also positively correlated with time from transplantation to biopsy (τ = 0.246, pFDR = 0.002; n = 123), but not with recipient age at transplantation. No significant association with transplant number was observed for either antibody. In group comparisons, anti-PAR1 and anti-PAR2 concentrations did not differ significantly according to recipient sex or donor type. In contrast, both anti-PAR1 and anti-PAR2 concentrations differed according to post-transplant timing when analyzed using cut-offs of ≤6 months versus >6 months and ≤12 months versus >12 months (**Table 3**).

**Table 3:** Correlation of anti-PAR1 and anti-PAR2 antibody concentrations with clinical characteristics and post-transplant timing.

Analysis / variable	Anti-PAR1	Anti-PAR2
Correlation analyses		
Recipient age at transplantation	τ = -0.110; p = 0.073; pFDR = 0.097; n = 123	τ = -0.177; p = 0.004; pFDR = **0.005**; n = 123
Time from transplantation to biopsy	τ = 0.246; p < 0.001; pFDR = **0.002**; n = 123	τ = 0.310; p < 0.001; pFDR = **0.001**; n = 123
Transplant number at biopsy	τ = -0.123; p = 0.166; pFDR = 0.166; n = 121	τ = -0.025; p = 0.741; pFDR = 0.741; n = 121
Group comparisons		
Donor type	Living donor: 5.22 (3.75–8.03); deceased donor: 5.25 (3.42–8.12); U = 1060; p = 0.519	Living donor: 5.45 (3.28–9.91); deceased donor: 4.73 (2.99–7.71); U = 1088; p = 0.399
Recipient sex	Female: 6.27 (4.30–7.83); male: 4.57 (3.31–9.05); U = 1996; p = 0.207	Female: 5.60 (3.17–7.71); male: 4.55 (2.99–8.50); U = 1809; p = 0.779
Time from transplantation to biopsy: ≤6 months vs >6 months	U = 2131; **p < 0.001**	U = 2327; **p < 0.001**
Time from transplantation to biopsy: ≤12 months vs >12 months	U = 2336; **p = 0.001**	U = 2557; **p < 0.001**

Kendall’s τ coefficients are shown. pFDR values were adjusted using the Benjamini–Hochberg false discovery rate procedure.

Bold values indicate statistically significant associations after FDR correction (pFDR < 0.05).

### 3.6 Anti-PAR autoantibodies and kidney allograft survival

The exploratory primary landmark analysis included 117 recipients with a functioning graft and a positive follow-up time after serum sampling. During follow-up, 54 death-censored graft-loss events occurred. Recipients with anti-PAR2 concentrations above the cohort median had a higher hazard of graft loss than recipients with concentrations below the median (HR = 2.136, 95% CI, 1.202–3.795; Cox p = 0.010; log-rank p = 0.008). In contrast, the association for anti-PAR1 concentrations above versus below the cohort median did not reach statistical significance (HR = 1.660, 95% CI, 0.960–2.870; Cox p = 0.070; log-rank p = 0.070).

When analyzed as continuous variables, anti-PAR2 was not significantly associated with graft loss (HR per 1 U/mL = 1.018, 95% CI, 0.995–1.041; p = 0.135). The association for continuous anti-PAR1 concentration was borderline (HR per 1 U/mL = 1.027, 95% CI, 1.000–1.055; p = 0.050).

A sensitivity analysis additionally included five recipients with no positive follow-up time after the index date, for whom graft loss was coded at time zero. This analysis included 122 recipients and 59 graft-loss events. The association between anti-PAR2 concentrations above the median and graft loss remained statistically significant (HR = 2.071, 95% CI, 1.199–3.577; Cox p = 0.009; log-rank p = 0.008). Anti-PAR1 concentrations above the median were also associated with graft loss in the sensitivity analysis (HR = 1.777, 95% CI, 1.048–3.012; Cox p = 0.033; log-rank p = 0.030).

Because the findings differed according to antibody parameterization and, for anti-PAR1, between the primary and sensitivity analyses, the graft survival results should be regarded as exploratory. The proportional hazards assumption was not violated in any model, and no statistically significant evidence of non-linearity was identified for the continuous antibody variables. Full results and model diagnostics are provided in **Supplementary Figure 2** and **Supplementary Tables 5** and **11**.

## 4 Discussion

To our knowledge, this study represents an initial clinical evaluation of circulating anti-PAR1 and anti-PAR2 autoantibodies in adult kidney transplant recipients undergoing clinically indicated allograft biopsy. The principal finding was a weak but consistent association between higher anti-PAR2 concentrations and several histological features of chronic allograft injury, including interstitial fibrosis, tubular atrophy, arteriolar hyalinosis, and total inflammation. The corresponding effect sizes were modest, with Kendall’s τ values ranging from approximately 0.19 to 0.23. In contrast, anti-PAR1 concentrations were not significantly associated with individual Banff lesion scores after correction for multiple testing. Neither antibody showed a statistically significant association with rejection phenotypes. In the exploratory landmark analysis, anti-PAR2 concentrations above the cohort median were associated with a higher hazard of death-censored graft loss, although this association was not significant when anti-PAR2 was analyzed continuously. These findings suggest that anti-PAR2 may be related to chronic allograft injury, but the cross-sectional and hypothesis-generating nature of the study precludes causal or definitive clinical conclusions.

The association between anti-PAR2 and chronic Banff lesions is biologically plausible. PAR2 is expressed by renal epithelial, endothelial, mesangial, and inflammatory cells, and experimental studies have linked PAR2 signaling to cytokine release, tubular injury, fibroblast activation, extracellular matrix deposition, cellular senescence, and renal fibrosis (12, 13, 17–19). If functionally active, anti-PAR2 autoantibodies might modify PAR2-dependent signaling and contribute to inflammatory and profibrotic pathways. Alternatively, they may arise secondarily in response to chronic tissue injury, altered receptor expression, or increased receptor exposure, thereby representing a marker of cumulative graft remodeling rather than a direct pathogenic mediator. Because receptor activation, antibody function, and tissue PAR2 expression were not assessed, these mechanisms remain speculative.

Both anti-PAR1 and anti-PAR2 concentrations increased with time from transplantation to biopsy, and the association was stronger for anti-PAR2. Because chronic Banff lesions also accumulate over time, post-transplant duration may partly account for the observed histological associations. Whether anti-PAR2 autoantibodies precede tissue remodeling or develop as a consequence of established injury cannot be determined from a single cross-sectional measurement.

Neither antibody was significantly associated with an AMR component, a TCMR component, or microvascular inflammation. These negative findings should be interpreted cautiously because only 23 recipients had an AMR component and only three had isolated AMR. In addition, contemporaneous HLA-DSA results were not systematically available, preventing reliable stratification of DSA-positive and DSA-negative antibody-mediated injury. The observed associations therefore cannot be interpreted as independent of HLA-DSA status, and the present data do not exclude a relationship between anti-PAR autoantibodies and selected forms of antibody-mediated injury.

The absence of significant anti-PAR1 histopathological associations contrasts with previous mechanistic studies showing PAR1-dependent endothelial activation by patient-derived immunoglobulins in kidney allograft vasculopathy and scleroderma renal crisis (14–16). This discrepancy may reflect differences between circulating antibody concentration and biological activity, including agonistic or antagonistic function, epitope specificity, immunoglobulin subclass, affinity, or receptor-activating capacity.

The exploratory landmark analysis indicated poorer death-censored graft survival among recipients with anti-PAR2 concentrations above the cohort median, and this association remained significant in the sensitivity analysis. However, anti-PAR2 was not significantly associated with graft loss when analyzed as a continuous variable. Moreover, dichotomization at the cohort median does not represent a clinically validated positivity or prognostic threshold. The survival finding should therefore be interpreted cautiously and requires confirmation in an independent cohort. For anti-PAR1, the median-dichotomized association was not significant in the primary analysis but reached statistical significance in the sensitivity analysis, further indicating limited robustness.

This study has several limitations. It was retrospective, single-center, and restricted to recipients undergoing clinically indicated biopsies, which limits generalizability to stable transplant populations. The sample size was limited for less frequent phenotypes, particularly AMR. Antibody concentrations were measured at a single time point, and standardized contemporaneous data on HLA-DSA, cumulative immunosuppressive exposure, donor characteristics, previous borderline or inconclusive rejection episodes, recurrent kidney disease, coexisting biopsy lesions, and detailed causes of graft loss were incomplete or unavailable. Therefore, residual confounding cannot be excluded. Chronic Banff lesions are nonspecific and may reflect alloimmune injury, calcineurin inhibitor toxicity, recurrent or *de novo* kidney disease, infection, ischemic injury, aging, or other processes. No centralized histopathological reassessment was performed, including specific evaluation for striped interstitial fibrosis and tubular atrophy. The graft survival analyses were exploratory and univariable, and the association observed after median-based dichotomization was not reproduced when anti-PAR2 was modeled continuously. Furthermore, the study did not assess antibody function or include a healthy or non-transplant reference group, and no clinically validated positivity threshold has been established for anti-PAR1 or anti-PAR2 in kidney transplant recipients.

The study also has several strengths, including detailed Banff lesion scoring, component-based rejection phenotyping, continuous antibody analysis, correction for multiple testing, landmark survival analyses aligned with the timing of antibody measurement, sensitivity analyses, and evaluation of regression model assumptions. The observed histopathological pattern is also biologically consistent with experimental evidence implicating PAR2 signaling in renal inflammation and fibrosis.

In conclusion, higher anti-PAR2, but not anti-PAR1, concentrations showed weak associations with interstitial fibrosis, tubular atrophy, arteriolar hyalinosis, and total inflammation in kidney transplant recipients undergoing clinically indicated biopsy. Neither antibody was significantly associated with active rejection phenotypes. In an exploratory landmark analysis, anti-PAR2 concentrations above the cohort median were associated with poorer death-censored graft survival, although no significant association was observed when anti-PAR2 was analyzed continuously. Anti-PAR2 may represent an adjunctive indicator of chronic allograft injury, but the present data do not establish a pathogenic role or a clinically validated prognostic threshold. Prospective studies incorporating serial measurements, functional assays, contemporaneous HLA-DSA assessment, and standardized clinical and histological evaluation are required.

## Data Availability

The original contributions presented in the study are included in the article/**Supplementary Material**. Further inquiries can be directed to the corresponding authors.
