# Supplementary material for: Anti-PAR2 autoantibodies are associated with chronic histological injury in kidney transplant recipients
Source: Front Immunol. 2026 Aug 20;17:1923916. doi: 10.3389/fimmu.2026.1923916 (PMC13538870; doi:10.3389/fimmu.2026.1923916)
Supplement: Supplementary file 1 [file SupplementaryFile1.docx]

Supplementary Material

# Supplementary Table S1. R packages and functions used for statistical analyses.

| Analysis step | R package(s) | Main function(s) used |
| --- | --- | --- |
| Assessment of normality | stats, ggplot2 | shapiro.test; stat_qq; stat_qq_line |
| Descriptive statistics | stats | median; quantile; table; prop.table |
| Correlation analyses and assessment of tied ranks | stats | cor.test |
| Benjamini–Hochberg correction (FDR) | stats | p.adjust |
| Non-parametric comparisons between groups | stats | wilcox.test; kruskal.test |
| Dunn post hoc analysis | rstatix | dunn_test |
| Kaplan–Meier survival analysis | survival, survminer | Surv; survfit; ggsurvplot |
| Log-rank test | survival | survdiff |
| Cox proportional hazards regression | survival | coxph |
| Assessment of proportional hazards assumption | survival | cox.zph |
| Logistic regression analyses | stats | glm |
| Influential observations | stats | cooks.distance |
| Multicollinearity | car | vif |
| Linearity of the logit | ggplot2 | geom_smooth |
| Model discrimination (C-index) | survival | concordance |
| Model comparison | stats | anova; AIC |

# Supplementary Figure S1. Q-Q plots assessing normality of recipient age at transplantation, time from transplantation to biopsy, follow-up time, anti-PAR1 concentration, and anti-PAR2 concentration.

## Supplementary Table S2. Assessment of normality using the Shapiro-Wilk test for selected group comparisons.

| **Clinical variable** | **anti-PAR1** | **anti-PAR2** |
| --- | --- | --- |
| Donor type | Living donor (19): W = 0.603; p < 0.001 Deceased donor (102): W = 0.689;  p < 0.001 | Living donor (19): W = 0.683; p < 0.001 Deceased donor (102): W = 0.615;  p < 0.001 |
| Recipient Sex | Female (45): W = 0.589; p < 0.001 Male (78): W = 0.624; p < 0.001 | Female (45): W = 0.576; p < 0.001 Male (78): W = 0.619; p < 0.001 |
| AMR component | present (23): W = 0.607; p < 0.001 absent (97): W = 0.620; p < 0.001 | present (23): W = 0.623; p < 0.001 absent (97): W = 0.616; p < 0.001 |
| TCMR component | present (77): W = 0.605; p < 0.001 absent (43): W = 0.642; p < 0.001 | present (77): W = 0.613; p < 0.001 absent (43): W = 0.580; p < 0.001 |

## Supplementary Table S3. Percentage of tied ranks for variables included in the correlation analysis between anti-PAR1 and anti-PAR2 antibody concentrations and Banff lesion scores.

| **Parameter** | **Percentage of tied ranks** |
| --- | --- |
| Anti-PAR1 antibody concentration | 0.0% |
| Anti-PAR2 antibody concentration | 0.0% |
| Interstitial inflammation | 95.0% |
| Tubulitis | 94.1% |
| Arteritis | 97.5% |
| Glomerulitis | 94.1% |
| Peritubular capillaritis | 94.1% |
| Interstitial fibrosis | 94.1% |
| Tubular atrophy | 94.1% |
| Fibrous intimal thickening | 94.9% |
| Transplant glomerulopathy | 93.8% |
| Mesangial matrix expansion | 95.2% |
| Arteriolar hyalinosis | 95.8% |
| Total inflammation | 94.1% |
| Inflammation in areas of interstitial fibrosis and tubular atrophy (i-IFTA) | 94.0% |
| C4d deposition in peritubular capillaries | 95.0% |

## Supplementary Table S4. Percentage of tied ranks for variables included in the correlation analysis between anti-PAR1 and anti-PAR2 antibody concentrations and clinical variables.

| **Parameter** | **Percentage of tied ranks** | **n** |
| --- | --- | --- |
| Anti-PAR1 antibody concentration | 0.0% | 123 |
| Anti-PAR2 antibody concentration | 0.0% | 123 |
| Age at transplantation | 58.5% | 123 |
| Time from transplantation to biopsy | 10.6% | 123 |
| Time to graft loss or last follow-up | 0.0% | 123 |
| Transplant number at biopsy | 97.5% | 121 |

**Supplementary Figure S2. Kaplan–Meier curves and assessment of the functional form of continuous antibody concentrations in the primary landmark analysis.**

Panels A and B show death-censored kidney allograft survival from the time of serum sampling according to anti-PAR1 and anti-PAR2 concentrations dichotomized at the cohort medians. Panels C and D show Martingale residuals plotted against continuous anti-PAR1 and anti-PAR2 concentrations, respectively, with locally smoothed curves.

**Supplementary Table S5. Assessment of Cox proportional hazards model assumptions in the primary landmark analysis.**

| **Model** | **PH assumption p** | **Linearity assessment p** |
| --- | --- | --- |
| Anti-PAR1, continuous | 0.433 | 0.531 |
| Anti-PAR2, continuous | 0.097 | 0.108 |
| Anti-PAR1, High vs Low | 0.349 | Not applicable |
| Anti-PAR2, High vs Low | 0.372 | Not applicable |

The proportional hazards assumption was assessed using scaled Schoenfeld residuals. No statistically significant violation was detected in any model. For continuous antibody concentrations, the functional form was assessed by comparing the linear Cox model with a model incorporating a natural cubic spline with three degrees of freedom using a likelihood ratio test. The spline models did not provide a significantly better fit, supporting the use of linear terms. PH, proportional hazards.

## Supplementary Figure S3. Cook distance and LOESS plots used to assess influential observations and linearity of the logit in logistic regression models for the AMR component.

Cook distance plot

LOESS plots

**Supplementary Table S6.** **Comparison of anti-PAR1 and anti-PAR2 concentrations according to rejection-related phenotypes.**

| **Analysis** | **anti-PAR1** | **anti-PAR2** |
| --- | --- | --- |
| MVI− vs MVI+ | U = 1677; p = 0.520 | U = 1587; p = 0.265 |
| AMR component present vs absent | U = 848; p = 0.135 | U = 897; p = 0.246 |
| TCMR component present vs absent | U = 1722; p = 0.718 | U = 1828; p = 0.347 |

U, Mann–Whitney U statistic; MVI, microvascular inflammation; AMR, antibody-mediated rejection; TCMR, T-cell-mediated rejection.

## Supplementary Table S7. Logistic regression analyses.

| Model / analysis | Variable | Effect estimate | p |
| --- | --- | --- | --- |
| Univariable rejection model | Anti-PAR1 | OR = 1.017  (95% CI 0.962-1.067) | 0.499 |
| Univariable rejection model | Anti-PAR2 | OR = 1.023  (95% CI 0.979-1.065) | 0.262 |
| AMR prediction, main model | Anti-PAR1 | OR = 1.005 | 0.879 |
| AMR prediction, main model | Anti-PAR2 | OR = 1.022 | 0.372 |
| AMR prediction, sensitivity model | Anti-PAR1 | OR = 0.989  (95% CI 0.879-1.079) | 0.822 |
| AMR prediction, sensitivity model | Anti-PAR2 | OR = 1.000  (95% CI 0.913-1.070) | 0.999 |

OR, odds ratio; CI, confidence interval.

## Supplementary Table S8. Assessment of multicollinearity and model discrimination in logistic regression models.

| **Analysis** | **VIF** | **C-index** |
| --- | --- | --- |
| Prediction of AMR component, main analysis | anti-PAR1: 1.34; anti-PAR2: 1.34 | 0.572 |
| Prediction of AMR component, sensitivity analysis | anti-PAR1: 1.12; anti-PAR2: 1.12 | 0.575 |

## Supplementary Table S9. Exploratory comparison of anti-PAR1 and anti-PAR2 concentrations across detailed histopathological rejection diagnoses.

| **Histopathological diagnosis** | **n** | **anti-PAR1 [U/mL], median (Q1-Q3)** | **anti-PAR2 [U/mL], median (Q1-Q3)** |
| --- | --- | --- | --- |
| AMR | 3 | 7.54 (4.94–8.44) | 5.15 (2.99–6.00) |
| Borderline changes for TCMR | 28 | 3.66 (2.88–6.43) | 2.97 (1.92–4.19) |
| Mixed rejection | 20 | 6.99 (4.50–8.63) | 5.73 (4.54–8.81) |
| No rejection | 40 | 5.38 (3.99–7.71) | 6.11 (3.57–8.37) |
| TCMR | 29 | 4.93 (4.03–10.45) | 4.65 (3.66–10.20) |
| Kruskal–Wallis H | - | 8.51 | 12.82 |
| df | - | 4 | 4 |
| p | - | 0.075 | 0.012 |

*This analysis was considered exploratory because of marked imbalance in diagnostic group sizes, particularly the low number of pure AMR cases.*

## Supplementary Table S10. Dunn post hoc analysis with Holm correction for anti-PAR2 concentrations across detailed histopathological rejection diagnoses.

| **Group comparison** | **Z** | **Adjusted p-value** |
| --- | --- | --- |
| AMR vs Borderline for TCMR | 0.319 | 1.000 |
| AMR vs Mixed rejection | -1.090 | 1.000 |
| Borderline for TCMR vs Mixed rejection | -2.968 | 0.030 |
| AMR vs No rejection | -0.870 | 1.000 |
| Borderline for TCMR vs No rejection | -2.899 | 0.034 |
| Mixed rejection vs No rejection | 0.564 | 1.000 |
| AMR vs TCMR | -0.874 | 1.000 |
| Borderline for TCMR vs TCMR | -2.732 | 0.050 |
| Mixed rejection vs TCMR | 0.499 | 1.000 |
| No rejection vs TCMR | -0.039 | 0.969 |

### Supplementary Table S11. Landmark analyses of death-censored kidney allograft survival from the time of serum sampling.

**Panel A. Primary landmark analysis**

| **Model** | **N** | **Events** | **HR** | **95% CI** | **Cox p** | **Log-rank p** | **PH assumption p** |
| --- | --- | --- | --- | --- | --- | --- | --- |
| Anti-PAR1, continuous, per 1 U/mL | 117 | 54 | 1.027 | 1.000–1.055 | 0.050 | — | 0.433 |
| Anti-PAR2, continuous, per 1 U/mL | 117 | 54 | 1.018 | 0.995–1.041 | 0.135 | — | 0.097 |
| Anti-PAR1, High vs Low | 117 | 54 | 1.660 | 0.960–2.870 | 0.070 | 0.070 | 0.349 |
| Anti-PAR2, High vs Low | 117 | 54 | 2.136 | 1.202–3.795 | 0.010 | 0.008 | 0.372 |

**Panel B. Sensitivity analysis including recipients with graft loss coded at time zero**

| **Model** | **N** | **Events** | **HR** | **95% CI** | **Cox p** | **Log-rank p** | **PH assumption p** |
| --- | --- | --- | --- | --- | --- | --- | --- |
| Anti-PAR1, continuous, per 1 U/mL | 122 | 59 | 1.024 | 0.998–1.051 | 0.069 | — | 0.320 |
| Anti-PAR2, continuous, per 1 U/mL | 122 | 59 | 1.017 | 0.995–1.040 | 0.132 | — | 0.120 |
| Anti-PAR1, High vs Low | 122 | 59 | 1.777 | 1.048–3.012 | 0.033 | 0.030 | 0.640 |
| Anti-PAR2, High vs Low | 122 | 59 | 2.071 | 1.199–3.577 | 0.009 | 0.008 | 0.340 |

The primary landmark analysis included 117 recipients with a functioning graft and positive follow-up time after serum sampling. The sensitivity analysis additionally included five recipients with no positive follow-up time after the index date, for whom graft loss was coded at time zero. Antibody concentrations were analyzed continuously and after dichotomization at the cohort medians: 5.33 U/mL for anti-PAR1 and 4.79 U/mL for anti-PAR2. Hazard ratios in the dichotomized analyses compare the High group with the Low group. The proportional hazards assumption was assessed using scaled Schoenfeld residuals and was not violated in any model. HR, hazard ratio; CI, confidence interval; PH, proportional hazards**.**
